# Promoting effect of neutrophils on lung tumorigenesis is mediated by CXCR2 and neutrophil elastase

**DOI:** 10.1186/1476-4598-12-154

**Published:** 2013-12-09

**Authors:** Lei Gong, Amber M Cumpian, Mauricio S Caetano, Cesar E Ochoa, Maria Miguelina De la Garza, Daniel J Lapid, Seyedeh Golsar Mirabolfathinejad, Burton F Dickey, Qinghua Zhou, Seyed Javad Moghaddam

**Affiliations:** 1 Departments of Pulmonary Medicine, The University of Texas M.D. Anderson Cancer Center, 1515 Holcombe Boulevard, Unit 1100, Houston, TX 77030, USA; 2 Tianjin Lung Cancer Institute, Tianjin Medical University General Hospital, Tianjin Medical University, Tianjin, China; 3 Department of Esophageal Cancer, Key Laboratory of Prevention and Therapy, Tianjin Medical University Cancer Institute and Hospital, National Clinical Research Center for Cancer, Tianjin, China

**Keywords:** Neutrophil, Elastase, Lung cancer, Inflammation, CXCR2, K-ras

## Abstract

**Background:**

Tumor cells produce various cytokines and chemokines that attract leukocytes. Leukocytes can amplify parenchymal innate immune responses, and have been shown to contribute to tumor promotion. Neutrophils are among the first cells to arrive at sites of inflammation, and the increased number of tumor-associated neutrophils is linked to poorer outcome in patients with lung cancer.

**Results:**

We have previously shown that COPD-like airway inflammation promotes lung cancer in a K-ras mutant mouse model of lung cancer (CC-LR). This was associated with severe lung neutrophilic influx due to the increased level of neutrophil chemoattractant, KC. To further study the role of neutrophils in lung tumorigenesis, we depleted neutrophils in CC-LR mice using an anti-neutrophil antibody. This resulted in a significant reduction in lung tumor number. We further selectively inhibited the main receptor for neutrophil chemo-attractant KC, CXCR2. Similarly, this resulted in suppression of neutrophil recruitment into the lung of CC-LR mice followed by significant tumor reduction. Neutrophil elastase (NE) is a potent elastolytic enzyme produced by neutrophils at the site of inflammation. We crossed the CC-LR mice with NE knock-out mice, and found that lack of NE significantly inhibits lung cancer development. These were associated with significant reduction in tumor cell proliferation and angiogenesis.

**Conclusion:**

We conclude that lung cancer promotion by inflammation is partly mediated by activation of the IL-8/CXCR2 pathway and subsequent recruitment of neutrophils and release of neutrophil elastase. This provides a baseline for future clinical trials using the IL-8/CXCR2 pathway or NE inhibitors in patients with lung cancer.

## Background

Lung cancer is the leading cause of cancer death worldwide accounting for 29% of all male and 26% of all female cancer deaths in 2012 [1]. Cigarette smoking (CS) is the principal cause of lung cancer, and CS induced lung cancer is characterized by a deregulated inflammatory microenvironment [2]. In addition, the association between chronic obstructive pulmonary disease (COPD), an inflammatory disease of the lung, and lung cancer has been demonstrated in population-based studies [3,4]. Smokers with COPD have an increased risk of lung cancer compared to smokers with comparable cigarette exposure but without COPD [5,6]. Importantly, among former smokers with COPD, even following withdrawal of cigarette smoke, inflammation persists and lung function continues to deteriorate as does the increased risk of lung cancer [7]. These facts suggest a strong link between airway inflammation and lung cancer promotion.

Tumor cells produce various cytokines and chemokines that attract leukocytes including neutrophils, dendritic cells, macrophages, lymphocytes and mast cells [6]. It is becoming increasingly clear that tumor-associated neutrophils (TANs) play a major role in cancer promotion [8]. During immune responses, neutrophils are among the first cells to arrive at sites of inflammation. The increased number of TANs is linked to poorer outcomes in patients with bronchioloalveolar carcinoma [9], and many patients with advanced cancer show high levels of blood neutrophils [10]. Furthermore, in histopathologic specimens of distal lung and in bronchoalveolar lavage fluid (BALF) from COPD patients, neutrophils are prominent [11,12], and shown to cause COPD progression [13]. We have previously established a COPD-like mouse model of airway inflammation induced by repetitive exposure to an aerosolized lysate of non-typeable *Haemophilus influenzae* (NTHi) [11], which is the most common bacterial colonizer of airways in COPD patients [14]. Then we showed that this type of airway inflammation promotes lung cancer in a K-ras mutant mouse model of lung cancer (CC-LR) [15]. This was associated with severe neutrophilic influx due to an increased level of neutrophil chemoattractant, KC, which was partially inhibited by using a natural non-specific anti-inflammatory agent, curcumin, and resulted in significant tumor suppression [16]. Therefore, we further dissected the role of neutrophils in lung tumorigenesis by selectively targeting neutrophils, its chemokine receptor (CXCR2) and its specific enzyme (neutrophil elastase). Neutrophil depletion, CXCR2 inhibition, and lack of neutrophil elastase (NE) all resulted in significant tumor reduction in our K-ras mutant mouse model of lung cancer.

## Results

### Neutrophil depletion inhibits lung cancer promotion

To test the effect of neutrophil depletion on lung cancer development, we treated the CC-LR mice with mLy-6G Ab 5 mg/kg i.p. twice a week. Two groups (N = 8) of 10-week-old CC-LR mice were treated with mLy-6G Ab for 4 weeks, with one of these groups exposed to the NTHi lysate once a week for 4 weeks for induction of a COPD-type inflammatory lung phenotype. Two other (N = 8) groups of mice were treated with isotype control while one of them was exposed to NTHi lysate. All groups were sacrificed one day after the fourth NTHi exposure.

**Figure 1:**
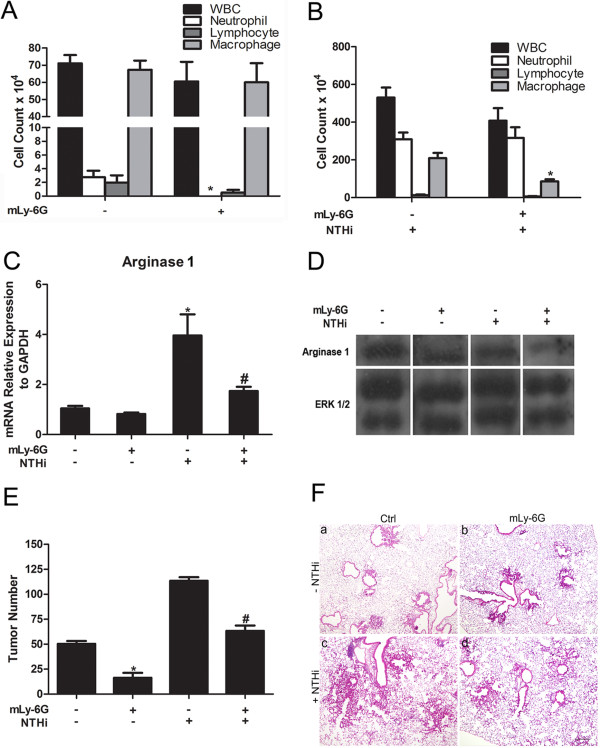
**Effect of treatment with anti-neutrophil antibody on lung inflammation and tumor promotion. (A)** Total and lineage-specific leukocyte number in BALF of CC-LR mice treated or non-treated with mLy-6G Ab at the age of 14 weeks (mean ± SE; * = P ≤ 0.05 for CC-LR vs CC-LR plus mLy-6G). **(B)** Total and lineage-specific leukocyte number in BALF of NTHi-exposed CC-LR mice treated or non-treated with mLy-6G Ab collected 1 day after last NTHi aerosol exposure at the age of 14 weeks (mean ± SE; * = P ≤ 0.05 for CC-LR vs CC-LR plus mLy-6G with NTHi exposure). **(C)** Real-time Q-PCR expression analysis of arginase 1 on the RNA extracted from whole lung tissue (normalized to GAPDH expression level, mean ± SE; * = P ≤ 0.05 for CC-LR vs CC-LR with NTHi exposure; # = P ≤ 0.05 for CC-LR with NTHi exposure vs CC-LR with NTHi exposure plus mLy-6G treatment). **(D)** Western blot analysis of arginase 1 on the protein extracted from whole lung tissue. **(E)** Lung surface tumor number after mLy-6G Ab treatment in NTHi exposed or non-exposed 14-week-old CC-LR mice. (mean ± SE; * = P ≤ 0.05 for CC-LR vs CC-LR plus mLy-6G treatment; # = P ≤ 0.05 for CC-LR with NTHi exposure vs CC-LR with NTHi exposure plus mLy-6G treatment). **(F)** Histopathological appearance of lung tissue after treatment with mLy-6G Ab in NTHi exposed or non-exposed CC-LR mice. (4× magnification, scale bar = 50 mm, applicable to all panels).

We and others have shown that expression of K-ras^G12D^ within the airway epithelium of mice induces the production of chemokines which leads to the accumulation of inflammatory cells, particularly macrophages and neutrophils, within the lung [15,17,18]. In the BALF of non-NTHi exposed Ab treated CC-LR mice, the total white blood cells decreased mostly due to complete depletion of neutrophils by the mLy-6G Ab (Figure 1A). The macrophage and lymphocyte counts were slightly reduced as well, because the mLy-6G Ab can non-specifically affect Gr-1^+^ monocytes/macrophages and lymphocyte subpopulations [19]. Surprisingly, the mLy-6G Ab was not able to completely deplete the neutrophils from the BALF of CC-LR mice after repetitive NTHi exposure, while the macrophages had a 2.4 reduction (Figure 1B).

Secreted cytokines and chemokines could both cause the recruitment of leukocytes and also help to identify the leukocyte phenotypes. Treatment with mLy-6G Ab in CC-LR mice non-exposed or exposed to NTHi resulted in a reduction in the level of neutrophil chemoattractant, KC in BALF (Table 1 and data not shown) which is consistent with reduced numbers of inflammatory cells in the BALF. CCL2 and CCL5 are phenotypic markers for TANs [20], which are protumorigenic (N2 phenotype). mLy-6G Ab inhibited these N2 type chemokine particularly CCL5 secretions (Table 1 and data not shown). Low iNOS and high arginase 1 expressions are two other important indicators of N2 type neutrophils [20-22]. Real-time Q-PCR analysis of the RNA extracted from the whole lung of CC-LR mice showed that treatment with mLy-6G Ab reduced the relative expression of arginase 1 in presence or absence of NTHi exposure (Figure 1C), while it had the opposite effect on relative expression of iNOS (Additional file 1: Figure S1A). This was associated with decreased protein expression of arginase 1 in western blot (WB) analysis of the whole lung from CC-LR mice treated with mLy-6G Ab (Figure 1C). Co-staining of lung tissue with Ly6G and arginase 1Abs showed the expression of arginase 1 in neutrophils (Additional file 1: Figure S1B). Levels of other essential proinflammatory cytokines (IL-6, IL-17, TGF-β) were slightly suppressed by mLy-6G Ab (Table 1 and data not shown). These results indicate that mLy-6G Ab could have an effect on tumor promotion by inhibiting neutrophil infiltration into the tumor microenvironment. Furthermore, mLy-6G Ab might be able to edit neutrophils phenotype from a pro-tumor phenotype (N2) toward an anti-tumor phenotype (N1).

**Table 1:** Cytokines and chemokines in bronchoalveolar fluid of CC-LR mice

**Cytokine**	Ctrl*	**+mLy-6G**	**+SBZ**
IL-6	4.66 ± 1.73	1.31 ± 1.39	1.34 ± 1.02
TGF-β	303.31 ± 99.50	210.55 ± 148.65	154.60 ± 134.40
IL-17	1.47 ± 1.95	0.55 ± 0.66	ND^†^
KC	9.48 ± 4.40	3.25 ± 1.57	5.41 ± 5.39
CCL5/RANTES	2.78 ± 0.73	0.58 ± 0.69	0.93 ± 0.26

*All data are means (±SEM), and there are no significant statistical differences (P>0.05).

^†^ND, not detected.

The effects of the neutrophil depletion using mLy-6G Ab on lung tumor promotion was analyzed by determining the number of tumors visible on the pleural surface of the lungs from CC-LR mice. mLy-6G Ab significantly reduced the tumor numbers on the lung surface of CC-LR mice not exposed to NTHi lysate by ~68% (3.2-fold, 51 ± 2 without mLy-6G versus 16 ± 2 with mLy-6G) and after NTHi exposure by ~80% (4.9-fold, 108 ± 11 without mLy-6G versus 22 ± 2 with mLy-6G) (Figure 1E). Histopathologic analysis showed that mLy-6G Ab treatment prevented lung cancer progression, with most of the lung tumors remaining at the early stage with less inflammatory cell infiltration both in the presence and absence of NTHi exposure (Figure 1F).

**Figure 2:**
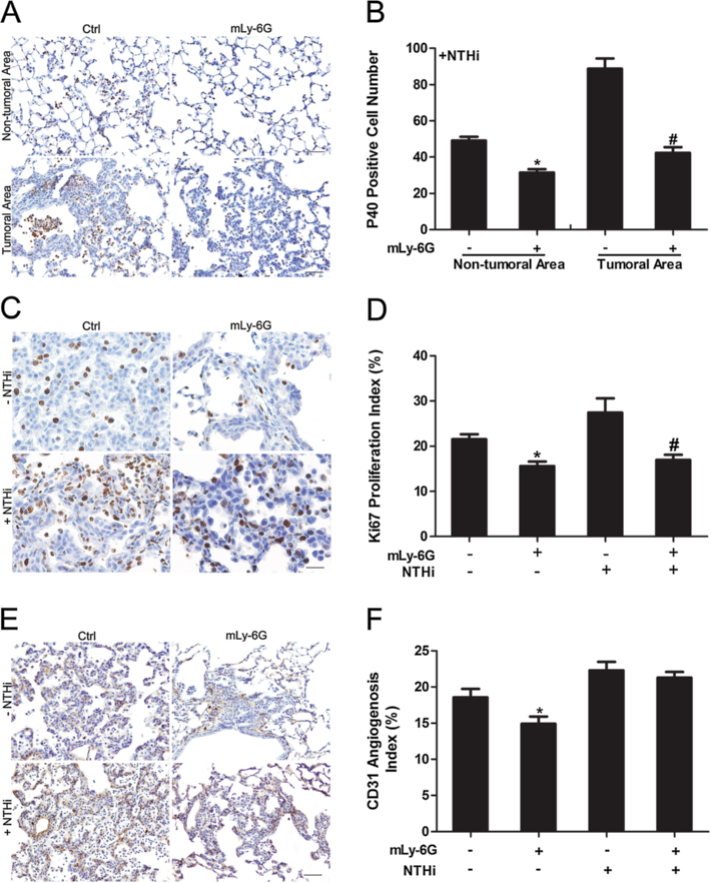
**Immunohistochemical analysis of lung tissue after treatment with anti-neutrophil antibody. (A)** Labeling with P40 Ab to detect infiltrating neutrophils in the non-tumoral area and tumoral area of the lung in CC-LR mice with or without mLy-6G treatment in presence of NTHi (20× magnification, scale bar = 50 mm, applicable to all panels). **(B)** Quantitative analysis of P40 positive staining in the non-tumoral area and tumoral area of the lung in CC-LR mice with or without mLy-6G treatment in the presence of NTHi (mean ± SE; * = P ≤ 0.05 for CC-LR with NTHi exposure vs CC-LR with NTHi exposure plus mLy-6G treatment in non-tumoral area; # = P ≤ 0.05 for CC-LR with NTHi exposure vs CC-LR with NTHi exposure plus mLy-6G treatment in tumoral area). **(C)** Representative photomicrographs of Ki-67 positive cells in lung tissue of CC-LR mice with or without mLy-6G treatment in the presence or absence of NTHi (40× magnification, scale bar = 50 mm, applicable to all panels). **(D)** Quantitative analysis of Ki-67 positive staining in lung tissue from CC-LR mice with or without mLy-6G in the presence or absence of NTHi exposure (mean ± SE; * = P ≤ 0.05 for CC-LR vs CC-LR plus mLy-6G treatment, # = P ≤ 0.05 for CC-LR with NTHi exposure vs CC-LR with NTHi exposure plus mLy-6G treatment). **(E)** Representative photomicrographs of CD31 positive cells in lung tissue of CC-LR mice with or without mLy-6G treatment in the presence or absence of NTHi (20× magnification, scale bar = 50 mm, applicable to all panels). **(F)** Quantitative analysis of CD31 positive staining in lung tissue of CC-LR mice with or without mLy-6G in the presence or absence of NTHi exposure (mean ± SE; * = P ≤ 0.05 for CC-LR vs CC-LR plus mLy-6G treatment).

We further stained for lung parenchymal and tumor infiltrating neutrophils using P40 Ab by IHC. We did not find any neutrophils infiltrating the lung of Ab treated non-NTHi exposed CC-LR mice compared to the lung of non-Ab treated mice (data not shown). NTHi exposure resulted in robust neutrophil infiltration which was significantly (1.6 fold) decreased by mLy-6G Ab treatment (Figure 2A). Interestingly, there were more neutrophils infiltrated in the tumor site than the normal lung tissue, and these neutrophils in the tumor site decreased 2.1 fold in response to treatment with mLy-6G Ab (Figure 2B).

In order to evaluate the mechanism of tumor suppression by mLy-6G Ab, we studied the rate of proliferation, angiogenesis and apoptosis. The relative number of cells showing positive staining for the cell proliferation marker, Ki-67, was measured in tumor tissues from the four groups by immunohistochemistry (Figure 2C). The mLy-6G Ab effectively suppressed the expression of Ki-67 in tumor tissues compared with non-Ab treated mice from both NTHi exposed and unexposed control groups (Figure 2D). Interestingly, in the NTHi exposed group, the infiltrating neutrophils also showed a high level of proliferation, suggesting the recruitment of immature neutrophils to the tumor site which are still going through proliferation (Figure 2D, Additional file 1: Figure S1C). This was suppressed with mLy-6G Ab treatment. In order to study the effect of mLy-6G Ab on angiogenesis, we performed the CD31 immunostaining, which is expressed on a large portion of endothelial cells [23]. CD31 expression followed the same pattern as Ki-67 (Figure 2E). The percentage of positive cells in the Ab treated group decreased (78%) compared to the control group in the absence of NTHi exposure. There was no significant change between the Ab treated and non-treated groups after NTHi exposure (Figure 2F). IHC analysis of tumor cell using cleaved caspase-3 marker did not show apoptosis among different groups (data not shown).

### Selective CXCR2 inhibition suppresses lung cancer promotion

**Figure 3:**
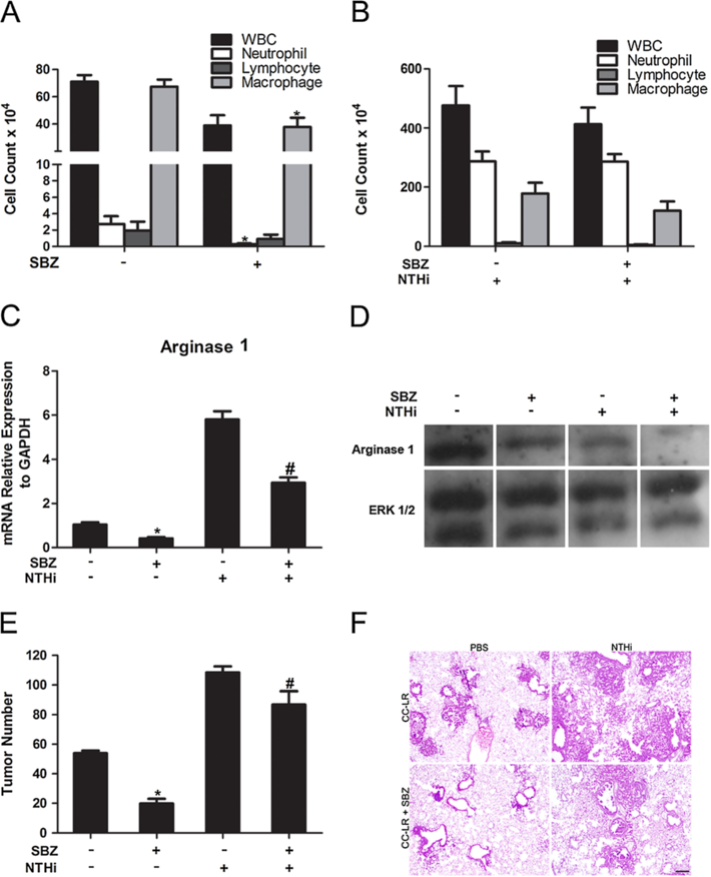
**Effect of treatment with a selective CXCR2 inhibitor on lung inflammation and tumor promotion. (A)** Total and lineage-specific leukocyte number in BALF of CC-LR mice treated or non-treated with SBZ (selective CXCR2 inhibitor) at the age of 14 weeks (mean ± SE; * = P ≤ 0.05 for CC-LR vs CC-LR plus SBZ). **(B)** Total and lineage-specific leukocyte number in BALF of NTHi-exposed CC-LR mice treated or non-treated with SBZ collected 1 day after last NTHi aerosol exposure at the age of 14 weeks. **(C)** Real-time Q-PCR analysis of RNA extracted from whole lung tissue for relative mRNA expression of arginase 1 (normalized to GAPDH expression level, mean ± SE; * = P ≤ 0.05 for CC-LR vs CC-LR plus SBZ treatment in figure D, # = P ≤ 0.05 for CC-LR with NTHi exposure versus CC-LR with NTHi exposure plus SBZ treatment). **(D)** Western blot analysis of arginase 1 on the protein extracted from whole lung tissue. **(E)** Lung surface tumor number after SBZ treatment in NTHi exposed or unexposed CC-LR mice. (mean ± SE; * = P ≤ 0.05 for CC-LR vs CC-LR plus SBZ treatment, # = P ≤ 0.05 for CC-LR with NTHi exposure vs CC-LR with NTHi exposure plus SBZ treatment). **(F)** Histopathological appearance of lung tissue after treatment with SBZ in NTHi exposed or unexposed CC-LR mice (4× magnification, scale bar = 50 mm, applicable to all panels).

As described above, KC is a very important chemo attractant for neutrophils. It could bind to the CXCR2 receptor, which will cause neutrophil recruitment [24], hence making it a good target for lung cancer treatment. We treated CC-LR mice with a selective CXCR2 inhibitor, SB332235Z (SBZ) 50 mg/kg orally, twice daily by gavage. Two groups (N = 8) of 10-week-old CC-LR mice were treated with SBZ for 4 weeks, with one of these groups exposed to NTHi lysate once a week for 4 weeks. Two other groups (N = 8) of mice were treated with vehicle control while one of them was exposed to NTHi lysate. All groups were sacrificed one day after the fourth NTHi exposure. Analysis of the BALF from SBZ treated non-NTHi exposed mice showed significant inhibition of neutrophil recruitment (Figure 3A). However, it did not effectively suppress neutrophil recruitment after 4 weeks of NTHi exposure (Figure 3B). Similarly, BALF KC levels also decreased by SBZ treatment in the non-NTHi exposed group, while remaining high in the NTHi exposed group (Table 1 and data not shown). Meanwhile, the levels of proinflammatory cytokines were suppressed by SBZ only in the non-NTHi group (Table 1 and data not shown). This was associated with decreased arginase 1 mRNA expression (Figure 3C) and increased iNOS mRNA expression (Additional file 2: Figure S2A) in the whole lung of CC-LR mice after SBZ treatment similar to what we found in samples treated with mLy-6G Ab. Furthermore, WB analysis of the whole lung from CC-LR mice treated with SBZ showed decreased protein expression of arginase 1 (Figure 3D).

Treatment with this selective CXCR2 antagonist reduced the tumor number on the lung surface of CC-LR mice not exposed to NTHi lysate by ~67% (3-fold, 54 ± 5 without SBZ versus 18 ± 2 with SBZ) and after NTHi exposure by ~23% (1.3-fold, 108 ± 11 without SBZ versus 83 ± 21 with SBZ) (Figure 3E). Histopathologic analysis of lung tissue from SBZ treated CC-LR mice showed that CXCR2 inhibition prevented lung cancer progression, with most of the lung tumors remaining at the early stage with less inflammatory cell infiltration both in the presence and absence of NTHi exposure (Figure 3F).

**Figure 4:**
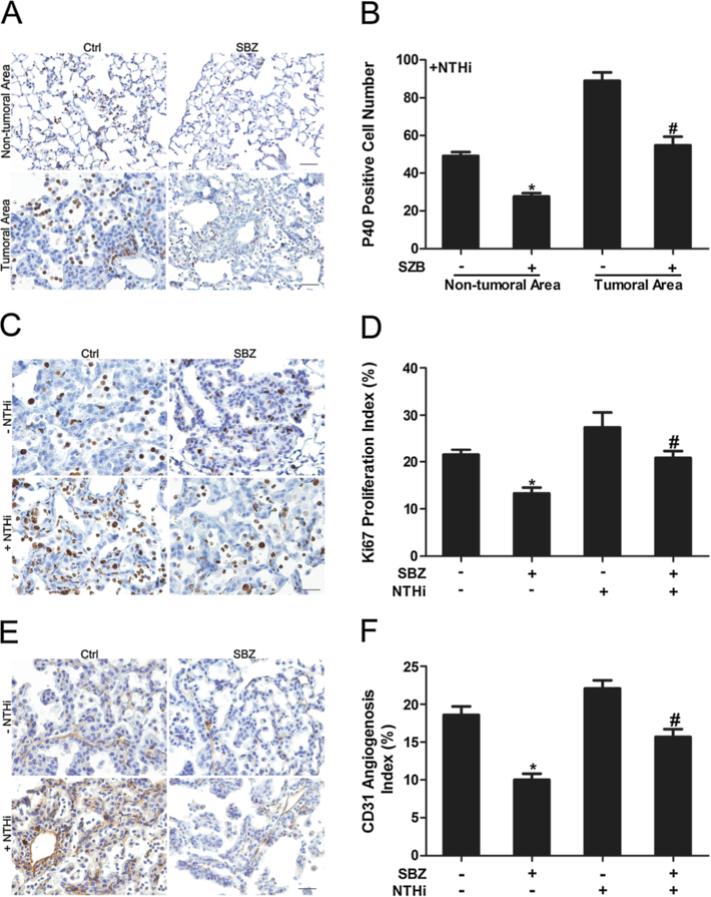
**Immunohistochemical analysis of lung tissue after treatment with selective CXCR2 inhibitor. (A)** Labeling with P-40 Ab to detect infiltrating neutrophils in non-tumoral area and in tumoral area in the lung of CC-LR mice with or without SBZ treatment in the presence of NTHi (20× magnification, scale bar = 50 mm, applicable to all panels). **(B)** Quantitative analysis of P-40 positive staining (mean ± SE; * = P ≤ 0.05 for CC-LR with NTHi exposure vs CC-LR with NTHi exposure plus SBZ treatment in non-tumoral area; # = P ≤ 0.05 for CC-LR with NTHi exposure vs CC-LR with NTHi exposure plus SBZ treatment in tumoral area). **(C)** Representative photomicrographs of Ki-67 positive cells in lung tissue of CC-LR mice with or without SBZ treatment in presence or absence of NTHi (40× magnification, scale bar = 50 mm, applicable to all panels). **(D)** Quantitative analysis of Ki-67 positive staining (mean ± SE; * = P ≤ 0.05 for CC-LR vs CC-LR plus SBZ treatment, # = P ≤ 0.05 for CC-LR with NTHi exposure vs CC-LR with NTHi exposure plus SBZ treatment). **(E)** Representative photomicrographs of CD31 positive cells in lung tissue of CC-LR mice with or without SBZ treatment in presence or absence of NTHi (20× magnification, scale bar = 50 mm, applicable to all panels). **(F)** Quantitative analysis of CD31 positive staining (mean ± SE; * = P ≤ 0.05 for CC-LR vs CC-LR plus SBZ treatment, # = P ≤ 0.05 for CC-LR with NTHi exposure vs CC-LR with NTHi exposure plus SBZ treatment).

IHC staining of the lung tissue from mice treated with SBZ with P40 antibody showed similar results as the mice treated with mLy-6G Ab (Figure 4A). Not many infiltrating neutrophils in the lung parenchyma were found in both non-NTHi groups. However, there was a 1.8 fold reduction in neutrophil infiltration by SBZ treatment in NTHi group (Figure 4B). There were more neutrophils found in the tumor site than the normal lung tissue, and infiltrating neutrophils in the tumor site decreased 1.6 fold in response to SBZ treatment (Figure 4B).

To evaluate the effect of SBZ on cell proliferation, Ki-67 was measured in tumor tissues from the four groups by immunohistochemistry (Figure 4C). CXCR2 inhibition with SBZ effectively suppressed the expression of Ki-67 in tumor tissues compared to both NTHi exposed and non-exposed control groups. Again, high numbers of proliferating neutrophils were found infiltrated in the tumor site (Figure 4D, Additional file 2: Figure S2B). To study the effect of CXCR2 inhibition on tumor angiogenesis, CD31 IHC staining was performed (Figure 4E). SBZ treatment resulted in a 53% decrease in CD31 expression in the non-NTHi exposed group, and a 68% decrease in CD31 expression in the NTHi exposed group compared to controls (Figure 4F). Similar to mLy-6G Ab treated groups, no apoptotic tumor cells was found after SBZ treatment (data not shown).

### Lack of neutrophil elastase (NE) inhibits lung cancer promotion

**Figure 5:**
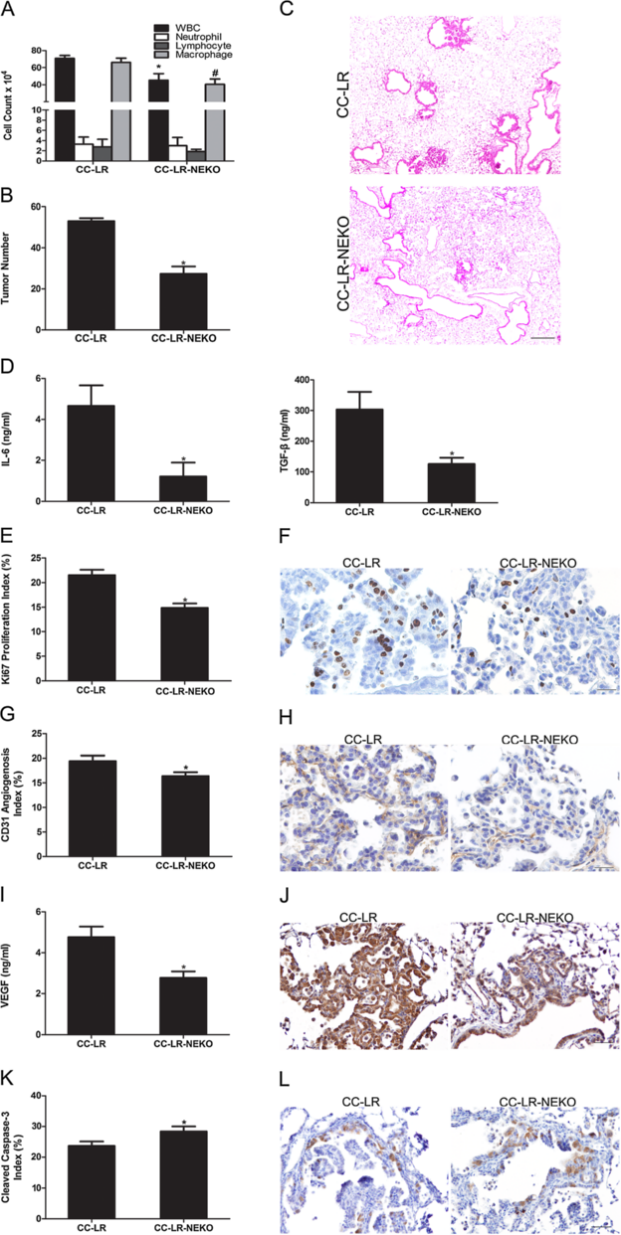
**Effect of neutrophil elastase (NE) depletion on lung tumor promotion. (A)** Total and lineage-specific leukocyte number in BALF of CC-LR mice and CC-LR-NEKO are shown (mean ± SE; * = P ≤ 0.05). **(B)** Lung surface tumor number of CC-LR and CC-LR-NEKO mice (mean ± SE; * = P ≤ 0.05 for CC-LR vs CC-LR-NEKO mice). **(C)** Histopathological appearance of lung tissue for CC-LR and CC-LR-NEKO (4× magnification, scale bar = 50 mm, applicable to all panels). **(D)** Level of IL-6 and TGF-β in BALF of CC-LR and CC-LR-NEKO mice (mean ± SE, * = P ≤ 0.05). **(E)** Quantitative analysis of Ki-67 positive staining in lung tissue of CC-LR and CC-LR-NEKO mice (mean ± SE; * = P ≤ 0.05). **(F)** Representative photomicrographs of Ki-67 positive cells in lung tissue of CC-LR and CC-LR-NEKO mice (40× magnification, scale bar = 50 mm, applicable to all panels). **(G)** Quantitative analysis of CD31 positive staining in lung tissue of CC-LR and CC-LR-NEKO mice (mean ± SE; * = P ≤ 0.05). **(H)** Representative photomicrographs of CD31 positive cells in lung tissue of CC-LR and CC-LR-NEKO mice (20× magnification, scale bar = 50 mm, applicable to all panels). **(I)** Expression level of the VEGF in BALF of CC-LR and CC-LR-NEKO mice (mean ± SE, * = P ≤ 0.05). **(J)** Representative photomicrograph of VEGF positive cells in lung tissue of CC-LR and CC-LR-NEKO mice (20× magnification, scale bar = 50 mm, applicable to all panels). **(K)** Quantitative analysis of cleaved-caspase-3 positive staining in lung tissue of CC-LR and CC-LR-NEKO mice (mean ± SE; *P ≤ 0.05). **(L)** Representative photomicrographs of cleaved-caspase-3 positive cells in lung tissue of CC-LR and CC-LR-NEKO mice (20× magnification, scale bar = 50 mm, applicable to all panels).

NE is an elastolytic enzyme, which is a potential regulator of the inflammatory process [25]. Many literatures verified that NE contributes to different stages of tumorigenesis from promotion to metastasis [26]. It also participates actively in the development of COPD [27] by activating proteolytic cascades. NE knock out (KO) mice were crossed with CC-LR mice to generate mice with activating mutant K-ras in the airway epithelium with global lack of NE (CC-LR-NEKO). Lack of NE slightly changed the BALF inflammatory cell component of the CC-LR mice, mostly macrophages (Figure 5A). However, it significantly inhibited lung cancer development by ~43% (1.7-fold, 53 ± 5 in CC-LR vs 31 ± 4 in CC-LR-NEKO) (Figure 5B). This was associated with decreased levels of IL-6 and TGF-β, (Figure 5D). There were no changes in tumor numbers and inflammatory cell infiltration of NTHi exposed CC-LR-NEKO mice (Additional file 3: Figure S3A, and S3B), suggesting roles for other recruited inflammatory cells, such as macrophages and their products (e.g. macrophage elastase) in promotion of lung cancer by COPD-type airway inflammation.

Histopathologic study of the lung from CC-LR-NEKO mice showed that absence of NE not only caused less tumor number but also it delayed lung cancer progression from early stage hyperplastic lesions to advanced adenoma and adenocarcinoma (Figure 5C). This was not the case for NTHi exposed CC-LR-NEKO mice compared to the NTHi exposed CC-LR mice (Additional file 3: Figure S3B). IHC analysis for tumor cell proliferation showed less Ki-67 expressing cells (69%) in the tumors from CC-LR-NEKO mice (Figure 5E and F). In addition, there was significant decrease (48%) in the number of CD31 positive cells in the CC-LR-NEKO compared to CC-LR group (Figure 5G and H), which indicates less angiogenesis in CC-LR-NEKO mice. Reduction of VEGF level in the BALF (Figure 5I) and less lung tissue VEGF positive staining (Figure 5J) in CC-LR-NEKO mice further verify the effects of NE on angiogenesis. Interestingly, we found a 1.2 fold increase in the number of cleaved-caspase-3 expressing cells in CC-LR-NEKO compared to control CC-LR mice (Figure 5K) with most of apoptosis happening in the early stage tumor sites (Figure 5L).

## Discussion

Tumor-induced inflammation is now a cancer hallmark, and it is considered an enabling characteristic due to its contributions to the acquisition of core hallmark capabilities [28,29]. Tumors are complex tissues composed of multiple distinct cell types that interact with each other. The recruited stromal and inflammatory cells, which form the tumor microenvironment, are active participants in tumorigenesis and significantly influence most of the cancer hallmarks in the malignant cancer cells. In summary, the presence of these stromal and inflammatory cells within the tumor microenvironment is associated with enhanced tumor progression, cancer cell growth and spread, angiogenesis, invasion and tumor immunosuppression [30].

Many environmental causes and risk factors for cancer are associated with chronic inflammation. According to epidemiological studies, up to 20% of cancers are linked to chronic infections and inflammation [31]. Cigarette smoking generates an inflammatory microenvironment which can promote lung cancer [2]. On the other hand, COPD, which is a disorder characterized by an abnormal local and systemic inflammatory response, is also strongly associated with lung cancer [32]. In histopathologic specimens of distal lung and in BALF from COPD patients, neutrophils and macrophages are prominent [33]. Neutrophils make up a significant portion of the inflammatory cell infiltrate found in a wide variety of murine models and human cancers [34,35]. They are among the first cells to arrive at sites of inflammation and, as mentioned before, the increased number of TANs is linked to poorer outcomes in patients with lung cancer. Neutrophils are present within the alveolar airspaces and within the tumor parenchyma during neoplastic development in a urethane-induced mouse model of lung cancer [36]. We have previously shown that bacterial (NTHi) lysate induced COPD-like airway inflammation promotes lung cancer in a K-ras mutant mouse model of lung cancer (CC-LR) [15]. This was associated with severe neutrophilic influx due to the increased level of neutrophil chemoattractant, KC. Furthermore, we and others found that K-ras mutation per se induces an inflammatory tumor microenvironment predominated by neutrophils and macrophages [15,17]. In this study, we provided more evidence to support the essential role for neutrophils in lung cancer promotion.

mLy-6G Ab is used to deplete neutrophils in murine disease models. It binds to Ly6G, which is present on neutrophils, and to Ly6C, which is expressed on neutrophils, dendritic cells, and subpopulations of lymphocytes and monocytes [19]. In our study, depletion of neutrophils by mLy-6G Ab suppressed lung tumor promotion which was associated with decreased tumor cell proliferation and angiogenesis. Similar to tumor associated macrophages (TAMs), TANs also can be divided into an antitumorigenic (N1) phenotype and a protumorigenic (N2) phenotype [20]. Analysis of cytokines and chemokines in BALF of CC-LR mice showed suppression of proinflammtory cytokines (IL-6, IL-17, TGF-β) by mLy-6G, with further inhibition of N2 type cytokines and molecules (CCL5, arginase 1). Surprisingly, mLy-6G Ab was not able to suppress the number of neutrophils in BALF of NTHi-exposed mice which can imply that there exists a pool of neutrophil that can be mobilized. However, mLy-6G Ab reduced the numbers of macrophages in the BALF and the number of neutrophils infiltrated in the lung parenchyma. Interestingly, many of these infiltrating neutrophils were found to be stained by Ki-67 in the tumor site, showing a proliferating state for these cells. These cells mostly express pro-tumor N2 phenotype markers and can be viewed as myeloid-derived suppressor cells (MDSCs), which represent a heterogeneous group of immature myeloid cells comprising macrophages, monocytes, neutrophils, and DCs [37] or myeloid progenitor cells at different stages of development that have been reported to be recruited along with PMNs to sites of inflammation and proliferate in tissue [38].

Interleukin-8 (IL-8), which is known as keratinocyte-derived chemokine (KC) in the murine, is a proinflammatory CXC chemokine associated with the neutrophil chemotaxis and degranulation [24]. It is also reported to be a transcriptional target of Ras Signaling, but its role in Ras-induced tumorigenesis has not been fully defined [39]. Expression and secretion of IL-8 by tumor cells enhance proliferation and neutrophil infiltration into the tumor [40-42]. According to our previous study, the expression of KC in the BALF of the CC-LR mice was higher compared to the WT mice [15]. The application of curcumin, which is a naturally occurring polyphenolic phytochemical isolated from the rhizome of the medical plant Curcuma longa, with non-specific anti-inflammatory effect, could suppress lung tumor promotion partly by suppressing KC production and subsequent inhibition of neutrophil recruitment to the lung [16]. In the current study, KC level was also suppressed by mLy-6G Ab in the non-NTHi-exposed group.

The protein encoded by the CXCR2 gene is a member of the G-protein couple receptor family. It is the receptor for IL-8. This receptor mediates neutrophil migration to sites of inflammation and is essential for neutrophil recruitment [43,44]. There is a remarkable up-regulation of CXCR2 in airway epithelial cells in COPD, and this correlates with the increased number of neutrophils in the airways [45]. Neutrophil recruitment into tumors also appears to be dependent on chemokines that bind to CXCR2 expressed by neutrophils [40]. Furthermore, CXC chemokines are potent promoters of angiogenesis, and mediate their angiogenic activity via signal-coupling of CXCR2 on endothelium [41,46,47]. It has recently been shown that CXCR2 expression in tumor cells is a poor prognostic factor and promotes invasion and metastasis in lung adenocarcinoma [48]. We found that inhibiting CXCR2 by a selective antagonist could efficiently suppress neutrophilic inflammation and lung tumor progression. This was associated with significant reduction in tumor cell proliferation and angiogenesis. This is similar to the findings from other groups showing that CXCR2 inhibition using a neutralizing antibody inhibits the progression of premalignant alveolar lesions by reducing vascular density in another mouse model of lung cancer [49]. Analysis of the cytokines and chemokines in the BALF of CC-LR mice showed that the secretion of pro-inflammatory cytokines (IL-6, IL-17, TGF-β) decreased after treatment with CXCR2 inhibitor with further reduction in levels of N2 type markers, CCL-5. This was associated with decreased expression of arginase 1, while the expression of iNOS increased. These results indicate that CXCR2 inhibition not only suppresses the recruitment of neutrophils but also may shift their differentiation from a N2 phenotype to a N1 phenotype. There was a much larger reduction in angiogenesis with CXCR2 inhibition as opposed to neutrophil depletion with mLy-6G Ab treatment. This could be due to inhibition of additive direct effect of CXCR2 axis activation on tumor endothelial cells that leads to less tumor angiogenesis.

Similar to mLy-6G Ab treatment, neutrophils in BALF of NTHi-exposed mice were not affected by the CXCR2 inhibitor, but it reduced the number of infiltrating neutrophils with MDSC/progenitor cell characteristics in the lung parenchyma and tumor sites. Failure of the anti-neutrophil Ab and CXCR2 inhibitor in inhibiting BALF neutrophilic influx in response to repetitive exposure to NTHi indicates involvement of other chemokines and inflammatory signaling pathways besides the IL-8/CXCR2 pathway in this phenomenon.

Products secreted from neutrophils, such as, reactive oxygen species and proteinases, have defined and specific roles in regulating tumor cell proliferation, angiogenesis, and metastasis [34]. Among them, NE is generally considered the major effector of neutrophil function, making up approximately 2% of the dry weight of a neutrophil [50]. It contributes to cigarette smoke-induced emphysema in mice [51]. NE KO mice are significantly protected from the development of CS-induced emphysema and recruitment of both neutrophils and macrophages. We have also found that lack of NE reduces lung macrophages and inhibits lung cancer promotion in our K-ras mutant mouse model indicating an essential promoting role for this enzyme in lung tumorigenesis. This is consistent with the study from Houghton et al., where they found the similar promoting effect for NE through degradation of IRS-1 in another mouse model of lung cancer [52]. NE exposure can activate PI3K/Akt pathway resulting in tumor cell proliferation and survival [52]. Lack of NE in our K-ras mutant model was associated with decreased proliferation and increased apoptosis of tumor cells which could be secondary to lack of PI3K/Akt pathway activity in absence of NE. Tumor regression was also accompanied with decreased levels of TGF-β similar to the finding by Kang-Yun et al., showing that NE up-regulates TGF-β gene expression and release via the MyD88/IRAK/NF-κB pathway [53]. It has been previously shown that TANs were involved in tumor angiogenesis by the production of proangiogenic factors such as VEGF and IL-8 [8], proteases such as MMPs [54] and elastases [55]. We have also found decreased expression of VEGF and numbers of CD31 positive cells in our K-ras mutant mouse with lack of NE, indicating a role for NE in tumor angiogenesis. It has also been shown that NE can cleave VEGF to generate a diffusible VEGF fragments that stimulate inflammatory cell recruitment and activation via VEGF receptor 1/Akt pathway [56] that could be considered as a tumor promoting mechanism of NE in our model. Similar to our findings Wada et al. have demonstrated that NE released from activated neutrophils stimulates the growth and progression of cancer cells by releasing the growth factors such as VEGF on the cell surface and that sivelestat, a specific NE inhibitor, blocks these processes [57].

It is known that K-ras induces the secretion of the cytokine IL-6 in various epithelial cell types including lung epithelial cells [58]. We have previously revealed increased levels of IL-6 and subsequent activation of the STAT3 pathway in our K-ras induced mouse model of lung cancer in absence and presence of COPD-like airway inflammation [59]. We further demonstrated that genetic ablation of IL-6 results in significant tumor suppression in this model in vivo, indicating an essential role for IL-6 in lung cancer promotion in this model. Therefore, reduced levels of IL-6 in response to neutrophil depletion, CXCR2 inhibition, or lack of NE could be considered as another mechanism of tumor regression. However, this could simply be a reflection of the reduced production of this cytokine from K-ras mutant cells due to reduction in tumor burden.

## Conclusions

Taken together, we confirmed that neutrophils play a very important role in the promotion of lung cancer which is strongly mediated through the IL-8/CXCR2 pathway and release of NE and development of a type 2 protumor microenvironment. This provides a baseline for future clinical trials and/or prevention trials using the IL-8/CXCR2 pathway or NE inhibitors in patients with lung cancer and high risk COPD patients.

## Materials and methods

### Animals

CCSP^Cre^/LSL-K-ras^G12D^ mice (CC-LR) were generated as previously described [15]. Briefly, this is a mouse generated by crossing a mouse harboring the LSL-K-ras^G12D^ allele with a mouse containing Cre recombinase inserted into the Clara cell secretory protein (CCSP) locus [15]. CC-LR mice were crossed with neutrophil elastase (NE) knock out (KO) mice [52,60], which were kindly provided by Dr. Steven Shapiro (University of Pittsburgh) to generate CC-LR-NEKO mice. All mice were housed in specific pathogen-free conditions and handled in accordance with the Institutional Animal Care and Use Committee of M. D. Anderson Cancer Center. Mice were monitored daily for evidence of disease or death.

### NTHi lysate aerosol exposure

A lysate of NTHi strain 12 was prepared as previously described [15], the protein concentration was adjusted to 2.5 mg/ml in phosphate buffered saline (PBS), and the lysate was frozen in 10 ml aliquots at −80°C. To deliver the lysate to mice by aerosol, a thawed aliquot was placed in an AeroMist CA-209 nebulizer (CIS-US, Bedford, MA) driven by 10 liter/min of room air supplemented with 5% CO_2_ for 20 min.

### Neutrophils depletion and CXCR2 inhibition

Neutrophils were depleted with the anti-neutrophils antibody (Ab), mLy-6G (clone: RB6-8C5; Bio X Cell, West Lebanon, NH, USA), 100ul at the concentration of 1 mg/ml per mouse, twice a week by intraperitoneal (i.p.) injection. CXCR2 was inhibited by using a selective inhibitor, SB332235Z, 50 mg/kg orally, twice daily by gavage (GSK, Brentford, UK).

### Histochemistry

The tracheas of euthanized mice were cannulated with PE-50 tubing and sutured into place. The lungs were infused with 10% buffered formalin (Sigma, St. Louis, MO) and then removed and placed in 10% buffered formalin for 18 hours. Tissues then were transferred to 75% ethanol, embedded in paraffin blocks, and sectioned at 5-mm thickness. The sections on glass slides were dried at 60°C for 15 minutes and then were deparaffinized and stained with hematoxylin and eosin (H&E) by incubating the tissues in Harris hematoxylin (Sigma) followed by serial eosin (Sigma) and graded ethanol steps. The H&E-stained slides were examined by a pathologist blinded to genotype and treatment, and the proliferative lesions of the lungs were evaluated in accordance with the recommendations of the Mouse Models of Human Cancer Consortium [61].

### Immunohistochemistry

Previously sectioned lung samples on slides were immunohistochemically (IHC) stained and evaluated for expression of Ki-67 (1:200; ab16667; abcam, MA, USA), P40 (1:100; MCA771G; Serotec, Oxford, UK), CD31 (1:10, 550274, BD Biosciences, CA, USA), VEGF (1:500; sc-152, Santa Cruz, CA, USA), cleaved caspase-3 (1:500, ab13847, abcam), Anti-Ly6g (1:100, ab25377, abcam), Arginase 1 (1:100, Thermo scientific, PA5-22009) DAPI (Signa, D9564-10MG), Alexa Fluor 488 Dye and Alexa Fluor 594 (Invitrogen). All antibodies reacted with mouse antigens. Heat-induced antigen retrieval was performed using 10 mmol/L of citrate buffer (pH 6) in a pressure cooker for 20 min. Endogenous peroxidase was quenched with 3% hydrogen peroxide for 30 min at room temperature. Blocking was performed with non-immune normal serum. Immunoreactivity for immunohistochemistry was detected using biotinylated IgG secondary antibodies specific for each primary antibody followed by incubation with ABC kit (Vector Laboratory, Burlingame, CA) for 30 min, and stained with diaminobenzidine chromogenic substrate for 4–10 min. Slides were counter-stained with Harris hematoxylin in for 30 sec, followed by dehydration and mounted with cytoseal 60 (ThermoFisher Scientific, Cheshire, UK). Images were obtained by an OLYMPUS BX 60 inverted microscope at ×4, ×20 or ×40 magnification with Image-Pro Plus, version 4.5.1.22. The numbers of labeled positive cells for any of these markers were quantitated as a fraction of total tumor nuclei per high power field (40×) in 10 fields from three mice of each group. Results were expressed as percentage of positive cells ± SE.

### Assessment of lung tumor burden and inflammation

On the first day after the final NTHi exposure, animals were euthanized by i.p. injection of a lethal dose of avertin (Sigma). In all mice (n = 8 per group per time point), lung surface tumor numbers were counted and then in some of them (n = 8 per group per time point), the lungs were prepared for histologic analysis as described above. In other mice (n = 8 per group per time point), bronchoalveolar lavage fluid (BALF) was obtained by sequentially instilling and collecting 2 aliquots of 1 mL PBS through a tracheostomy cannula. Total leukocyte count was determined using a hemacytometer, and cell populations were determined by cytocentrifugation of 300 mL of BALF followed by Wright-Giemsa staining. The remaining BALF (1,400 mL) was centrifuged at 1,250 × g for 10 min, and supernatants were collected and stored at −80°C.

### Cytokines and chemokines measurement

The levels of KC, TNF, IL-6, IL-10, IL-17A, CCL2, CCL5 and VEGF in the BALF were assessed using MCYTOMAG-70 K assay (Millipore, St Charles, MO), according to the manufacturer’s instructions). Due to reagent incompatibilities, TGF-β was assayed separately from the other cytokines using the TGFB-64 K-01 (Millipore, MO, USA) assay. Data were collected using a Luminex 100 (Luminex Corporation, TX, USA). Standard curves were generated using a 5-parameter logistic curve-fitting equation weighted by 1/y (StarStation V 2.0; Applied Cytometry Systems, CA, USA). Each sample reading was interpolated from the appropriate standard curve.

### Quantitative RT-PCR analysis

Total RNA was isolated from whole lung according to the TRIzol reagent protocol (Invitrogen, NY, USA) and purified by E.Z.N.A. total RNA kit I (OMEGA, GA, USA). Reverse transcription PCR was performed using the qScript cDNA SuperMix (Quanta biosciences, MD, USA). Real-time PCR was carried out according to a standard protocol using the PerfeCTaFast Mix II (Quanta) with the TaqMan probe for arginase 1 and iNOS (ABI, NY, USA), and products measured on an ABI Viia 7 PCR system (ABI). Relative expression of each gene was calculated and graphed for comparison among the treatment groups. GAPDH RNA was measured for reference.

### Western blot analysis

Total proteins were prepared from each group of mouse lungs. Lung samples were removed and immediately placed in RIPA buffer and a protease inhibitor mixture. The samples were then homogenized and centrifuged at 14000 g for 20 min at 4°C. The supernatants were collected as the total proteins. Equal amounts (40 μg) of the total proteins were boiled for 5 min in the presence of loading buffer, loaded on each lane, and separated by 10% SDS-PAGE. The gels were then transferred to nitrocellulose membranes. Equal amounts of protein loading for each lane was checked by Ponceau (Sigma Chemical Co., MO, USA) staining. The anti-arginase 1 (Thermo Scientific, clone PA5-22009, MA, USA) antibody was diluted to the concentration according to commercial recommendations (1:1000). Immunoreactive bands were detected with Pierce ECL Western Blotting Substrate (Pierce, IL, USA).

### Statistical methods

Summary statistics for cell counts in BALF, IHC positive cells in lung tissue, and mRNA expression were computed within treatment groups, and analysis of variance with adjustment for multiple comparisons was conducted to examine the differences between the control group and each of the treatment groups in the presence or absence of NTHi exposure. For tumor counts, comparisons of groups were made using Student’s t test. Differences were considered significant for P < 0.05.

## Abbreviations

COPD: Chronic obstructive pulmonary disease; NTHi: Non-typeable *Haemophilus influenzae*; PBS: Phosphate buffered saline; BALF: Bronchoalveolar lavage fluid; H&E: Hematoxylin and eosin; TANs: Tumor-associated neutrophils; NE: Neutrophil elastase; CS: Cigarette smoking; CXCR2: Chemokine (C-X-C motif) receptor 2; MDSCs: Myeloid-derived suppressor cells; IL-8: Interleukin-8.

## Competing interests

The authors declare that they have no competing interests.

## Authors’ contributions

LG carried out the mouse in vivo study including mLy6-G and CXCR2 injection, weekly NTHi exposure, and lung tissue extraction, and participated in preparing the figures and drafting the manuscript. AMC, MSC, CEO, and DJL participated in the mouse colony maintenance, genotyping, and lung tissue extraction and western blot analysis. MMD and SGM carried out the histopathology examination and analysis of the lung tissues and participated in preparing the figures. BFD and QZ participated in the design of the study and the drafting the manuscript. SJM designed the study, assessed lung tumor burden and inflammation, performed the statistical analysis, and participated in preparing the figures and drafting the manuscript. All authors read and approved the final manuscript.

## Supplementary Material

Additional file 1: Figure S1 (A) Real-time Q-PCR analysis of RNA extracted from whole lung tissue for relative mRNA expression of iNOS (normalized to GAPDH expression level, mean ± SE). (B) Representative immunofluorescence staining showing Ly6G + and Arginase 1+ double positive neutrophils after exposure to NTHi (Scale bar, 50 μm). (C) Representative immunofluorescence staining showing P40+ and Ki67+ double positive neutrophils with and without mLy-6G treatment after NTHi exposure (Scale bar, 50 μm). Click here for file

Additional file 2: Figure S2 (A) Real-time Q-PCR analysis of RNA extracted from whole lung tissue for relative mRNA expression of iNOS (normalized to GAPDH expression level, mean ± SE). (B) Representative immunofluorescence staining showing P40+ and Ki67+ double positive neutrophils with and without SBZ treatment after NTHi exposure (Scale bar, 50 μm). Click here for file

Additional file 3: Figure S3 (A) Lung surface tumor number in CC-LR and CC-LR-NEKO mice afxxter NTHi exposure (N = 4). (B) Total and lineage-specific leukocyte number in BALF of NTHi-exposed CC-LR and CC-LR-NEKO mice collected 1 day after last NTHi aerosol exposure at the age of 14 weeks (mean ± SE). (C) Histopathological appearance of lung tissue in CC-LR and CC-LR-NEKO after NTHi exposure. Click here for file

## References

[B1] Siegel R Naishadham D Jemal A Cancer statistics for Hispanics/Latinos, 2012 CA Cancer J Clin 2012 12 62

[B2] Spitz MR Gorlov IP Dong Q Wu X Chen W Chang DW Etzel CJ Caporaso NE Zhao Y Christiani DC Multistage analysis of variants in the inflammation pathway and lung cancer risk in smokers Cancer Epidemiol Biomarkers Prev 2012 12 1213 1221 10.1158/1055-9965.EPI-12-0352-T 22573796 PMC3487592

[B3] Shacter E Weitzman SA Chronic inflammation and cancer Oncology (Williston Park) 2002 12 217 226 229; discussion 230–212 11866137

[B4] Celli BR Chronic obstructive pulmonary disease and lung cancer: common pathogenesis, shared clinical challenges Proc Am Thorac Soc 2012 12 74 79 10.1513/pats.201107-039MS 22550249

[B5] Stellman SD Takezaki T Wang L Chen Y Citron ML Djordjevic MV Harlap S Muscat JE Neugut AI Wynder EL Smoking and lung cancer risk in American and Japanese men: an international case–control study Cancer Epidemiol Biomarkers Prev 2001 12 1193 1199 11700268

[B6] Coussens LM Werb Z Inflammation and cancer Nature 2002 12 860 867 10.1038/nature01322 12490959 PMC2803035

[B7] Shapiro SD End-stage chronic obstructive pulmonary disease: the cigarette is burned out but inflammation rages on Am J Respir Crit Care Med 2001 12 339 340 10.1164/ajrccm.164.3.2105072c 11500330

[B8] Fridlender ZG Albelda SM Tumor-associated neutrophils: friend or foe? Carcinogenesis 2012 12 949 955 10.1093/carcin/bgs123 22425643

[B9] Bellocq A Antoine M Flahault A Philippe C Crestani B Bernaudin JF Mayaud C Milleron B Baud L Cadranel J Neutrophil alveolitis in bronchioloalveolar carcinoma: induction by tumor-derived interleukin-8 and relation to clinical outcome Am J Pathol 1998 12 83 92 9422526 PMC1858104

[B10] Schmidt H Bastholt L Geertsen P Christensen IJ Larsen S Gehl J von der Maase H Elevated neutrophil and monocyte counts in peripheral blood are associated with poor survival in patients with metastatic melanoma: a prognostic model Br J Cancer 2005 12 273 278 10.1038/sj.bjc.6602702 16052222 PMC2361564

[B11] Moghaddam SJ Clement CG De la Garza MM Zou X Travis EL Young HW Evans CM Tuvim MJ Dickey BF *Haemophilus influenzae* lysate induces aspects of the chronic obstructive pulmonary disease phenotype Am J Respir Cell Mol Biol 2008 12 629 638 10.1165/rcmb.2007-0366OC 18096867 PMC2396243

[B12] Zhang X Zheng H Zhang H Ma W Wang F Liu C He S Increased interleukin (IL)-8 and decreased IL-17 production in chronic obstructive pulmonary disease (COPD) provoked by cigarette smoke Cytokine 2011 12 717 725 10.1016/j.cyto.2011.09.010 21996014

[B13] Gane J Stockley R Mechanisms of neutrophil transmigration across the vascular endothelium in COPD Thorax 2012 12 553 561 10.1136/thoraxjnl-2011-200088 21543441

[B14] Berenson CS Wrona CT Grove LJ Maloney J Garlipp MA Wallace PK Stewart CC Sethi S Impaired alveolar macrophage response to Haemophilus antigens in chronic obstructive lung disease Am J Respir Crit Care Med 2006 12 31 40 10.1164/rccm.200509-1461OC 16574934 PMC2662920

[B15] Moghaddam SJ Li H Cho SN Dishop MK Wistuba II Ji L Kurie JM Dickey BF Demayo FJ Promotion of lung carcinogenesis by chronic obstructive pulmonary disease-like airway inflammation in a K-ras-induced mouse model Am J Respir Cell Mol Biol 2009 12 443 453 10.1165/rcmb.2008-0198OC 18927348 PMC2660561

[B16] Moghaddam SJ Barta P Mirabolfathinejad SG Ammar-Aouchiche Z Garza NT Vo TT Newman RA Aggarwal BB Evans CM Tuvim MJ Curcumin inhibits COPD-like airway inflammation and lung cancer progression in mice Carcinogenesis 2009 12 1949 1956 10.1093/carcin/bgp229 19793800 PMC2783007

[B17] Ji H Houghton AM Mariani TJ Perera S Kim CB Padera R Tonon G McNamara K Marconcini LA Hezel A K-ras activation generates an inflammatory response in lung tumors Oncogene 2006 12 2105 2112 10.1038/sj.onc.1209237 16288213

[B18] Thatcher TH McHugh NA Egan RW Chapman RW Hey JA Turner CK Redonnet MR Seweryniak KE Sime PJ Phipps RP Role of CXCR2 in cigarette smoke-induced lung inflammation Am J Physiol Lung Cell Mol Physiol 2005 12 L322 L328 10.1152/ajplung.00039.2005 15833762 PMC2491909

[B19] Daley JM Thomay AA Connolly MD Reichner JS Albina JE Use of Ly6G-specific monoclonal antibody to deplete neutrophils in mice J Leukoc Biol 2008 12 64 70 17884993 10.1189/jlb.0407247

[B20] Fridlender ZG Sun J Kim S Kapoor V Cheng G Ling L Worthen GS Albelda SM Polarization of tumor-associated neutrophil phenotype by TGF-beta: “N1” versus “N2” TAN Cancer Cell 2009 12 183 194 10.1016/j.ccr.2009.06.017 19732719 PMC2754404

[B21] Mantovani A The yin-yang of tumor-associated neutrophils Cancer Cell 2009 12 173 174 10.1016/j.ccr.2009.08.014 19732714

[B22] O’Sullivan T Saddawi-Konefka R Vermi W Koebel CM Arthur C White JM Uppaluri R Andrews DM Ngiow SF Teng MW Cancer immunoediting by the innate immune system in the absence of adaptive immunity J Exp Med 2012 12 1869 1882 10.1084/jem.20112738 22927549 PMC3457735

[B23] Cartana T Saftoiu A Gruionu LG Gheonea DI Pirici D Georgescu CV Ciocalteu A Gruionu G Confocal laser endomicroscopy for the morphometric evaluation of microvessels in human colorectal cancer using targeted anti-CD31 antibodies PLoS One 2012 12 e52815 10.1371/journal.pone.0052815 23285192 PMC3532115

[B24] Waugh DJ Wilson C The interleukin-8 pathway in cancer Clin Cancer Res 2008 12 6735 6741 10.1158/1078-0432.CCR-07-4843 18980965

[B25] Xu Y Zhang J Han J Pan X Cao Y Guo H Pan Y An Y Li X Curcumin inhibits tumor proliferation induced by neutrophil elastase through the upregulation of alpha1-antitrypsin in lung cancer Mol Oncol 2012 12 405 417 10.1016/j.molonc.2012.03.005 22507634 PMC5528353

[B26] Sato T Takahashi S Mizumoto T Harao M Akizuki M Takasugi M Fukutomi T Yamashita J Neutrophil elastase and cancer Surg Oncol 2006 12 217 222 10.1016/j.suronc.2007.01.003 17320378

[B27] Hoenderdos K Condliffe A The Neutrophil in COPD: Too Little Too Late, or Too Much Too Soon? Am J Respir Cell Mol Biol 2013 10.1165/rcmb.2012-0492TR 23328639

[B28] Floor SL Dumont JE Maenhaut C Raspe E Hallmarks of cancer: of all cancer cells, all the time? Trends Mol Med 2012 12 509 515 10.1016/j.molmed.2012.06.005 22795735

[B29] Hanahan D Weinberg RA Hallmarks of cancer: the next generation Cell 2011 12 646 674 10.1016/j.cell.2011.02.013 21376230

[B30] Lorusso G Ruegg C The tumor microenvironment and its contribution to tumor evolution toward metastasis Histochem Cell Biol 2008 12 1091 1103 10.1007/s00418-008-0530-8 18987874

[B31] Mantovani A Allavena P Sica A Balkwill F Cancer-related inflammation Nature 2008 12 436 444 10.1038/nature07205 18650914

[B32] O’Callaghan DS O’Donnell D O’Connell F O’Byrne KJ The role of inflammation in the pathogenesis of non-small cell lung cancer J Thorac Oncol 2010 12 2024 2036 10.1097/JTO.0b013e3181f387e4 21155185

[B33] Jeffery PK Lymphocytes, chronic bronchitis and chronic obstructive pulmonary disease Novartis Found Symp 2001 12 149 161 discussion 161–148 11199094

[B34] Gregory AD Houghton AM Tumor-associated neutrophils: new targets for cancer therapy Cancer Res 2011 12 2411 2416 10.1158/0008-5472.CAN-10-2583 21427354

[B35] Carpagnano GE Palladino GP Lacedonia D Koutelou A Orlando S Foschino-Barbaro MP Neutrophilic airways inflammation in lung cancer: the role of exhaled LTB-4 and IL-8 BMC Cancer 2011 12 226 10.1186/1471-2407-11-226 21649887 PMC3130703

[B36] Redente EF Orlicky DJ Bouchard RJ Malkinson AM Tumor signaling to the bone marrow changes the phenotype of monocytes and pulmonary macrophages during urethane-induced primary lung tumorigenesis in A/J mice Am J Pathol 2007 12 693 708 10.2353/ajpath.2007.060566 17255336 PMC1851863

[B37] Sica A Porta C Morlacchi S Banfi S Strauss L Rimoldi M Totaro MG Riboldi E Origin and functions of Tumor-Associated Myeloid Cells (TAMCs) Cancer Microenviron 2012 12 133 149 10.1007/s12307-011-0091-6 21948460 PMC3399067

[B38] Kim MH Granick JL Kwok C Walker NJ Borjesson DL Curry FR Miller LS Simon SI Neutrophil survival and c-kit(+)-progenitor proliferation in Staphylococcus aureus-infected skin wounds promote resolution Blood 2011 12 3343 3352 10.1182/blood-2010-07-296970 21278352 PMC3069674

[B39] Wislez M Fujimoto N Izzo JG Hanna AE Cody DD Langley RR Tang H Burdick MD Sato M Minna JD High expression of ligands for chemokine receptor CXCR2 in alveolar epithelial neoplasia induced by oncogenic kras Cancer Res 2006 12 4198 4207 10.1158/0008-5472.CAN-05-3842 16618742

[B40] Tazzyman S Barry ST Ashton S Wood P Blakey D Lewis CE Murdoch C Inhibition of neutrophil infiltration into A549 lung tumors in vitro and in vivo using a CXCR2-specific antagonist is associated with reduced tumor growth Int J Cancer 2011 12 847 858 10.1002/ijc.25987 21328342

[B41] Lee YS Choi I Ning Y Kim NY Khatchadourian V Yang D Chung HK Choi D LaBonte MJ Ladner RD Interleukin-8 and its receptor CXCR2 in the tumour microenvironment promote colon cancer growth, progression and metastasis Br J Cancer 2012 12 1833 1841 10.1038/bjc.2012.177 22617157 PMC3364111

[B42] Han L Jiang B Wu H Wang X Tang X Huang J Zhu J High expression of CXCR2 is associated with tumorigenesis, progression, and prognosis of laryngeal squamous cell carcinoma Med Oncol 2012 12 2466 2472 10.1007/s12032-011-0152-1 22274915

[B43] Jones MR Simms BT Lupa MM Kogan MS Mizgerd JP Lung NF-kappaB activation and neutrophil recruitment require IL-1 and TNF receptor signaling during pneumococcal pneumonia J Immunol 2005 12 7530 7535 16301661 10.4049/jimmunol.175.11.7530 PMC2723739

[B44] Brandau S Trellakis S Bruderek K Schmaltz D Steller G Elian M Suttmann H Schenck M Welling J Zabel P Lang S Myeloid-derived suppressor cells in the peripheral blood of cancer patients contain a subset of immature neutrophils with impaired migratory properties J Leukoc Biol 2011 12 311 317 10.1189/jlb.0310162 21106641

[B45] Barnes PJ The cytokine network in chronic obstructive pulmonary disease Am J Respir Cell Mol Biol 2009 12 631 638 10.1165/rcmb.2009-0220TR 19717810

[B46] Belperio JA Keane MP Arenberg DA Addison CL Ehlert JE Burdick MD Strieter RM CXC chemokines in angiogenesis J Leukoc Biol 2000 12 1 8 10914483

[B47] Keeley EC Mehrad B Strieter RM CXC chemokines in cancer angiogenesis and metastases Adv Cancer Res 2010 12 91 111 20399957 10.1016/S0065-230X(10)06003-3 PMC3069502

[B48] Saintigny P Massarelli E Lin S Ahn YH Chen Y Goswami S Erez B O’Reilly MS Liu D Lee JJ CXCR2 expression in tumor cells is a poor prognostic factor and promotes invasion and metastasis in lung adenocarcinoma Cancer Res 2013 12 571 582 10.1158/0008-5472.CAN-12-0263 23204236 PMC3548940

[B49] Keane MP Belperio JA Xue YY Burdick MD Strieter RM Depletion of CXCR2 inhibits tumor growth and angiogenesis in a murine model of lung cancer J Immunol 2004 12 2853 2860 14978086 10.4049/jimmunol.172.5.2853

[B50] Belaaouaj A McCarthy R Baumann M Gao Z Ley TJ Abraham SN Shapiro SD Mice lacking neutrophil elastase reveal impaired host defense against gram negative bacterial sepsis Nat Med 1998 12 615 618 10.1038/nm0598-615 9585238

[B51] Shapiro SD Goldstein NM Houghton AM Kobayashi DK Kelley D Belaaouaj A Neutrophil elastase contributes to cigarette smoke-induced emphysema in mice Am J Pathol 2003 12 2329 2335 10.1016/S0002-9440(10)63589-4 14633606 PMC1892384

[B52] Houghton AM Rzymkiewicz DM Ji H Gregory AD Egea EE Metz HE Stolz DB Land SR Marconcini LA Kliment CR Neutrophil elastase-mediated degradation of IRS-1 accelerates lung tumor growth Nat Med 2010 12 219 223 10.1038/nm.2084 20081861 PMC2821801

[B53] Lee KY Ho SC Lin HC Lin SM Liu CY Huang CD Wang CH Chung KF Kuo HP Neutrophil-derived elastase induces TGF-beta1 secretion in human airway smooth muscle via NF-kappaB pathway Am J Respir Cell Mol Biol 2006 12 407 414 10.1165/rcmb.2006-0012OC 16690986

[B54] Shang K Bai YP Wang C Wang Z Gu HY Du X Zhou XY Zheng CL Chi YY Mukaida N Li YY Crucial involvement of tumor-associated neutrophils in the regulation of chronic colitis-associated carcinogenesis in mice PLoS One 2012 12 e51848 10.1371/journal.pone.0051848 23272179 PMC3525572

[B55] Mittendorf EA Alatrash G Qiao N Wu Y Sukhumalchandra P St John LS Philips AV Xiao H Zhang M Ruisaard K Breast cancer cell uptake of the inflammatory mediator neutrophil elastase triggers an anticancer adaptive immune response Cancer Res 2012 12 3153 3162 10.1158/0008-5472.CAN-11-4135 22564522 PMC3397251

[B56] Kurtagic E Jedrychowski MP Nugent MA Neutrophil elastase cleaves VEGF to generate a VEGF fragment with altered activity Am J Physiol Lung Cell Mol Physiol 2009 12 L534 L546 10.1152/ajplung.90505.2008 19136576 PMC2660218

[B57] Wada Y Yoshida K Tsutani Y Shigematsu H Oeda M Sanada Y Suzuki T Mizuiri H Hamai Y Tanabe K Neutrophil elastase induces cell proliferation and migration by the release of TGF-alpha, PDGF and VEGF in esophageal cell lines Oncol Rep 2007 12 161 167 17143494

[B58] Ancrile B Lim KH Counter CM Oncogenic Ras-induced secretion of IL6 is required for tumorigenesis Genes Dev 2007 12 1714 1719 10.1101/gad.1549407 17639077 PMC1920165

[B59] Ochoa CE Mirabolfathinejad SG Ruiz VA Evans SE Gagea M Evans CM Dickey BF Moghaddam SJ Interleukin 6, but not T helper 2 cytokines, promotes lung carcinogenesis Cancer Prev Res (Phila) 2011 12 51 64 10.1158/1940-6207.CAPR-10-0180 21098042 PMC3058282

[B60] Kaynar AM Houghton AM Lum EH Pitt BR Shapiro SD Neutrophil elastase is needed for neutrophil emigration into lungs in ventilator-induced lung injury Am J Respir Cell Mol Biol 2008 12 53 60 10.1165/rcmb.2007-0315OC 18276796 PMC2438448

[B61] Nikitin AY Alcaraz A Anver MR Bronson RT Cardiff RD Dixon D Fraire AE Gabrielson EW Gunning WT Haines DC Classification of proliferative pulmonary lesions of the mouse: recommendations of the mouse models of human cancers consortium Cancer Res 2004 12 2307 2316 10.1158/0008-5472.CAN-03-3376 15059877

