# Supplementary figures and images for: Promoting effect of neutrophils on lung tumorigenesis is mediated by CXCR2 and neutrophil elastase
Source: Mol Cancer. 2013 Dec 9;12:154. doi: 10.1186/1476-4598-12-154 (PMC3923587; doi:10.1186/1476-4598-12-154)

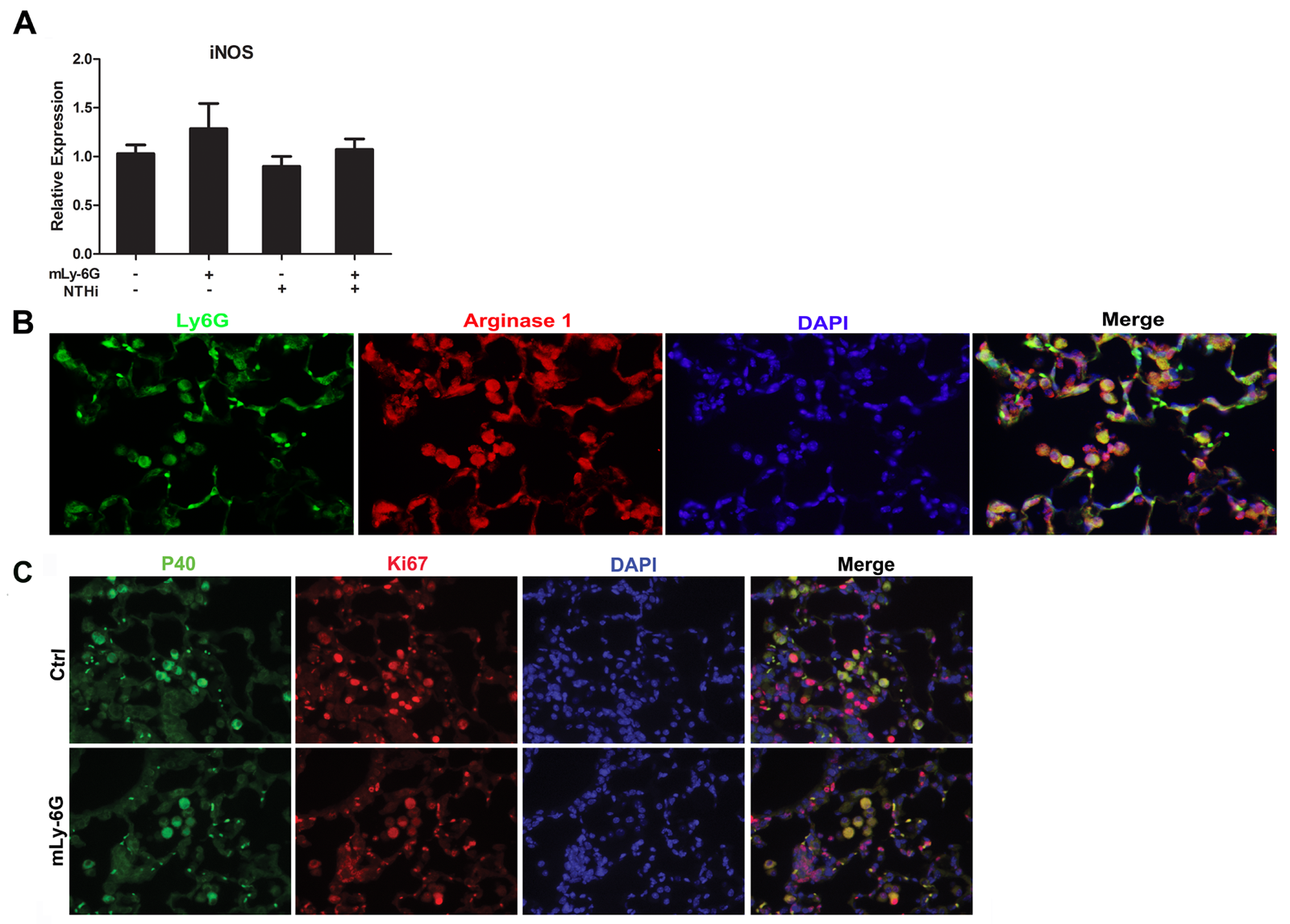

Supplement: Additional file 1: Figure S1 — (A) Real-time Q-PCR analysis of RNA extracted from whole lung tissue for relative mRNA expression of iNOS (normalized to GAPDH expression level, mean ± SE). (B) Representative immunofluorescence staining showing Ly6G + and Arginase 1+ double positive neutrophils after exposure to NTHi (Scale bar, 50 μm). (C) Representative immunofluorescence staining showing P40+ and Ki67+ double positive neutrophils with and without mLy-6G treatment after NTHi exposure (Scale bar, 50 μm). [file 1476-4598-12-154-S1.tiff]

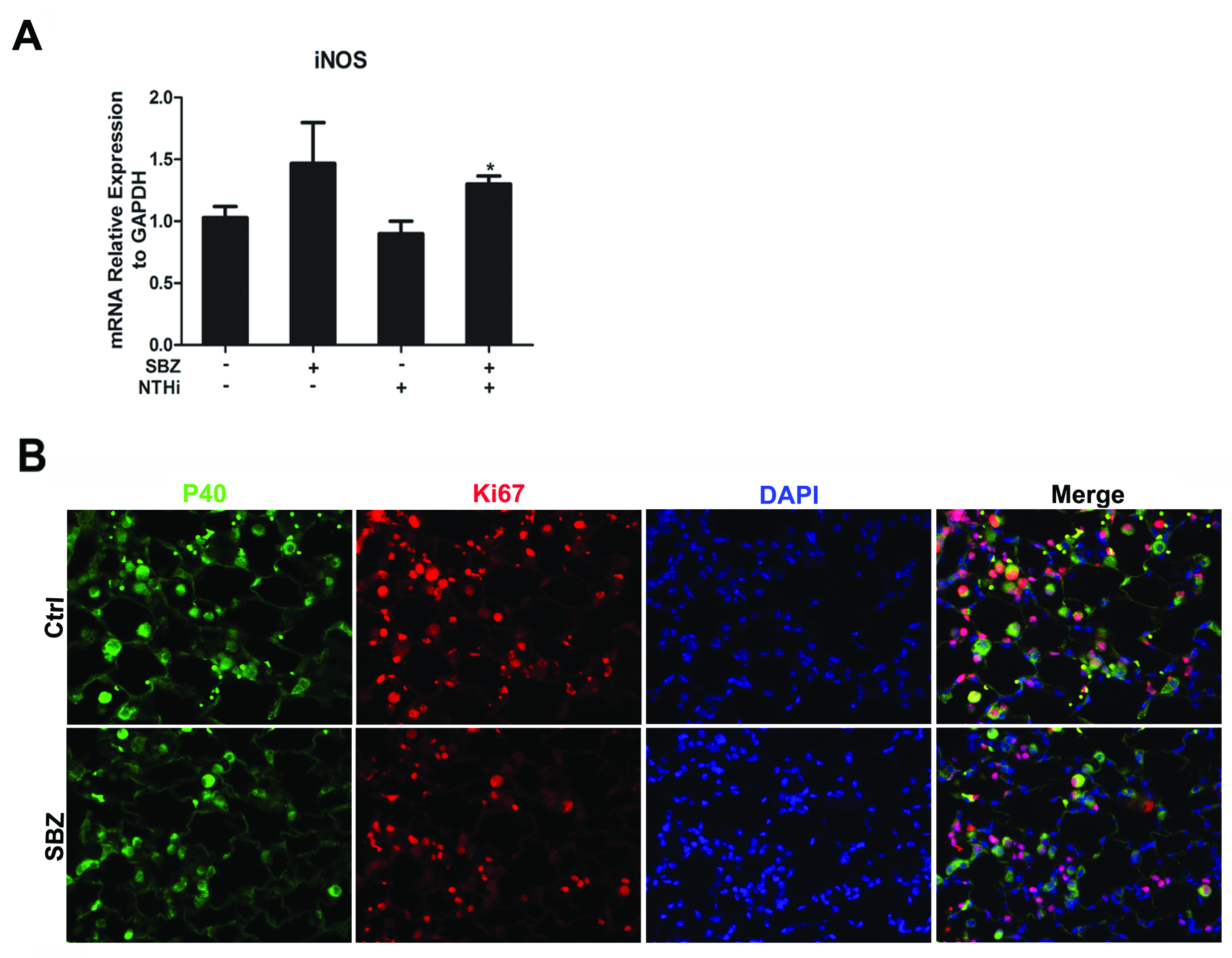

Supplement: Additional file 2: Figure S2 — (A) Real-time Q-PCR analysis of RNA extracted from whole lung tissue for relative mRNA expression of iNOS (normalized to GAPDH expression level, mean ± SE). (B) Representative immunofluorescence staining showing P40+ and Ki67+ double positive neutrophils with and without SBZ treatment after NTHi exposure (Scale bar, 50 μm). [file 1476-4598-12-154-S2.tiff]

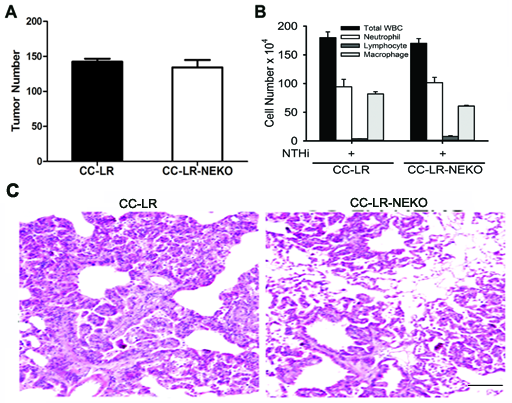

Supplement: Additional file 3: Figure S3 — (A) Lung surface tumor number in CC-LR and CC-LR-NEKO mice afxxter NTHi exposure (N = 4). (B) Total and lineage-specific leukocyte number in BALF of NTHi-exposed CC-LR and CC-LR-NEKO mice collected 1 day after last NTHi aerosol exposure at the age of 14 weeks (mean ± SE). (C) Histopathological appearance of lung tissue in CC-LR and CC-LR-NEKO after NTHi exposure. [file 1476-4598-12-154-S3.tiff]
